# Tracing the Trends of General Construction and Demolition Waste Research Using LDA Modeling Combined With Topic Intensity

**DOI:** 10.3389/fpubh.2022.899705

**Published:** 2022-05-25

**Authors:** Zezhou Wu, Peiying Xie, Jinming Zhang, Baojian Zhan, Qiufeng He

**Affiliations:** ^1^College of Civil and Transportation Engineering, Shenzhen University, Shenzhen, China; ^2^School of Political Studies, Nanjing Agricultural University, Nanjing, China

**Keywords:** construction and demolition waste, research trend, linear discriminant analysis, topic modeling, topic intensity

## Abstract

The study of construction and demolition waste (CDW) has attracted more and more attentions with the increasing CDW pollution caused by the large-scale infrastructure construction. This study used the Latent Dirichlet Allocation (LDA) combined with topic intensity to discover hot topics and development trends in the study area of CDW. First, the LDA was used for topic modeling to extract the existing topics from textual data. Second, the topic intensity was calculated for the extracted topics and the numerical values of the topic intensity represented the popularity of the topics. In this study, 4 topics were extracted from 1,849 relevant articles through the LDA modeling and topic intensity calculation. The results showed that the topic of “CDW management” had an upward trend. Topics such as “recycled aggregate,” “environmental impact,” and “study of CDW on soil” all showed a downward trend. The methods of this study can dig into the topics of CDW study and help scholars to engage in this field for better understanding the prevalence and evolution trends of these topics.

## Introduction

With the constant advancement of the construction industry, the problems that construction brings to the environment are becoming more and more obvious (1–3) and the daily construction and demolition waste (CDW) output of CDW is increasing daily (4–6). To improve the level of CDW, various countries are implementing a series of CDW measures, such as Sweden's implementation of the “Ordinance on Landfilling of Waste,” the Netherlands's “Environmental Protection Act,” and Japan's “Construction Material Recycling Law”(7). In this context, researchers and policymakers are required to accurately grasp the development trend of the field to support the formulation of national CDW-related policies (8, 9). Scientific measurement and text mining methods are used to sort out the study context from the massive articles and predict the research frontier that plays an extremely critical role in grasping the latest study trends of CDW.

In recent years, to grasp the research trend of CDW, relevant scholars have discussed the development trend of CDW from the perspective of the whole or partial field of CDW (10, 11). The majority of previous study used the method of literature measurement or the keywords combined with the method of scientific mapping analysis to predict the topic. In 2011, Yuan (12) selected 87 articles from 8 journals of CDW management during 2000–2009 as a sample library. The descriptive analysis, simulation/modeling, statistical analysis, and cost-benefit analysis were used to mine and analyze study topics and trends of CDW management. In 2017, Liu (13) adopted bibliometric analysis and imagined investigations were likewise applied in this article to assess the status and patterns of CDW study in view of articles from 2000 to 2016. In 2020, Li (14) used journal articles concerning the study and development of information technology in CDW management from 2000 to 2019 as the data source and used the method of scientific mapping analysis to analyze the study trend in the past 20 years. In addition, Ding (15) and Ranjbari (16) used topic modeling to disclose the study topic of CDW management. These existing review studies, however, only surveyed articles in the field of CDW management and also failed to investigate study trends in the field of CDW. As a result, the existing literature lacks an analysis of study hotspots in CDW. According to the above analysis, study reviews can be achieved through bibliometric analysis or topic modeling. However, most such study reviews mainly analyze journal, time, country, and author information and rarely analyze the content of the study, which limits the analysis of relevant study content. Among them, Latent Dirichlet Allocation (LDA) is an unsupervised topic model that can be used to analyze the topic importance of textual documents. To fill the study gap, this article used the LDA modeling combined with topic intensity to conduct an in-depth analysis of the main study topics of CDW and explored the development trend of each topic according to the average weight and topic intensity of different periods.

The LDA model is a generative probabilistic topic model. Given that the potential subject is the likelihood appropriation of a progression of words in the corpus, then, at that point, the record is the likelihood dispersion of a progression of expected themes. Unlike the overall bunching technique, the LDA permits a record to contain different themes simultaneously, so it is more suitable to extract study topics from scientific articles (17). At the earliest, Griffiths (18) used the LDA to extract the subject and subject change trend of the Proceedings of the National Academy of Sciences (PNAS) journal abstracts and used the Gibbs sampling algorithm to infer the LDA. Currently, the LDA has been used for the analysis of computer linguistics (19), library and information management (20), economics (21), and other fields (22–25), to understand the study status of a particular field by automatically mining study topics of a large number of articles. Hence, in this article, the LDA was used as the strategy for theme display. First, essential issues can be examined in CDW by determining the extent to which they have been addressed in CDW study. Second, information about the study trend of CDW can be extracted by studying how people's interest in the subject changes over time. Third, relatively neglected or marginalized topics can be explored and used to identify future study topics. To investigate the trend of CDW study, this study is based on the articles distributed within the Web of Science (WoS), trying to map the development of CDW study in the past 10 years (2011–2020). According to the average weight and topic intensity of different periods, it profoundly analyzes the main study topics of CDW and the development trend of each topic. The purpose of evaluating these topics is to study the research topics discussed by researchers in CDW. The text and abstract of the article are used as analysis objects by discussing its objectives, methods, and results. This article analyzes the use of point demonstration to extricate the principal subjects of the exploration and the distinctions of themes distributed in different periods to improve the accuracy of topic selection.

To sum up, this study extracts the topics of CDW articles with the help of the LDA classic model. It makes a detailed analysis of the content and intensity of each topic to deeply understand the study status of CDW in China from 2011 to 2020.

The next part of this article is organized as follows. Section 2 introduces the previous study work on the data source and implementation of the LDA-based topic modeling. Section 3 describes the analysis results. The last part is the conclusion.

## Data Source and Implementation of The Latent Dirichlet Allocation-Based Topic Modeling

### Data Source

**Figure 1:**
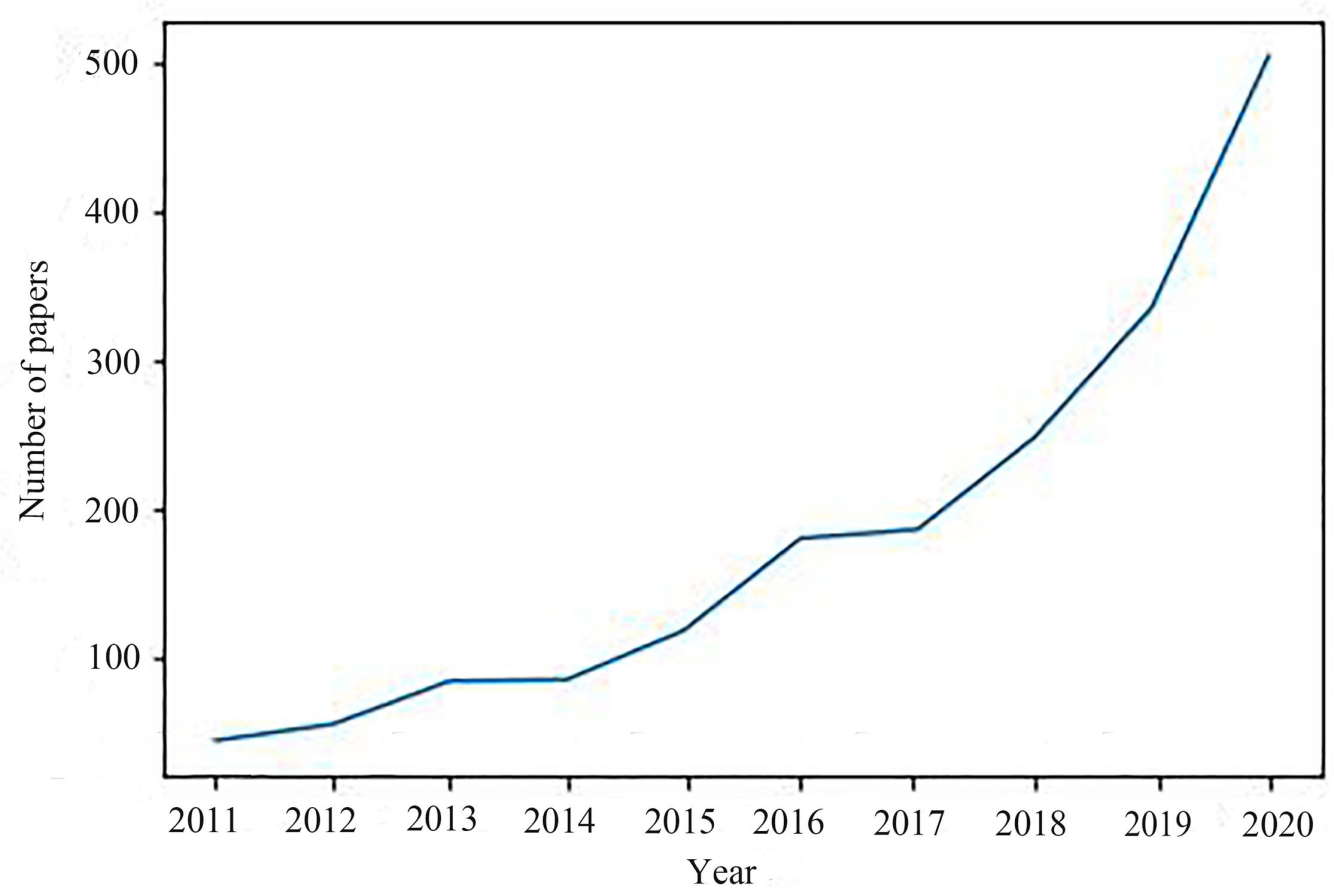
Number of articles in 2011–2020.

When retrieving from the WoS database, TS = “demolition and construction waste” or “C&D waste” or “C&D wastes” or “CDW” or “demolition waste” or “construction waste” (as the search type. At the same time, conference papers were excluded given that conference papers provide less data than journal articles. Finally, 1,849 articles related to the English language from 2011 to 2020 were collected. The annual distribution of 1,849 articles is shown in Figure 1, indicating that the overall number of articles in CDW study is steadily increasing.

### Data Preprocessing

The stage of preprocessing is exceptionally important to move forward the quality investigation of text data that is unstructured and information preprocessing is an activity that should be embraced, particularly within the investigation of web-based unstructured literary materials (26). The processing of data is mainly for identification dispensation, stop word removal, and morphological restoration of the above data. In this study, the processing of data is implemented and programmed in Python. In data processing, the smallest processing unit understood by the computer is a word, so text information must be identified. The identification here is to divide the sentence into a series of meaningful word segmentation.

Stop words removal is to remove meaningless words such as conjunctions, articles, and pronouns. The vocabulary made up of meaningless words is called the stop word list. This article removes the English stop words and adopts the stop word list of the Natural Language Toolkit (NLTK). The stop word list of the NLTK is relatively standard and complete, covering the stop word list in 22 languages. By passing language name parameters to the library's stop words constructor, stop words in different languages can be removed to improve the reliability of results. The lemmatization operation uses the setting and morphology to decide the variety of related words and uses different normalization rules to obtain connected roots based on the grammatical feature (27). In this study, the titles and abstracts of texts were extracted from 1,849 articles. All the text characters are changed into lowercase and any exceptional characters or stop words are deleted. Any grammatical marker, such as the plural form of a noun or the past form of a verb, can be converted to its general form, removing punctuation and unnecessary characters altogether. Some articles have two-letter combinations of words, such as focus group, requiring two-letter combinations and lexical recovery, as well as calling them sequentially.

### Latent Dirichlet Allocation-Based Topic Modeling Implementation

The choice of the number of topics is the first step in building a model. The topic of the LDA model is an abstract concept. According to different granularities, the topics in the corpus can be divided into various quantities of themes. The quantity of subjects in the LDA needs to be given in advance. In general, larger corpora represent more topics.

Furthermore, the LDA gives a bunch of words compared to themes based on the number of points. Hence, it is essential to investigate the appropriate number of themes. If not, it will affect the advantages and disadvantages of the entire topic model. Suppose that the selected value of the number of topics is too small. In this case, the ability of the model to describe the data will be limited. Instead, it will prolong the training time of the model and hinder the effect of data analysis. Perplexity is often used to determine the number of topics (28).

Moreover, although perplexity is widely used, it is not the best way to evaluate the number of topics because it does not consider the semantic association between word contexts. This study selects the ideal number of themes in light of the coherence score. It uses the method provided by Mallet to calculate a coherence score, depending on the level of co-event between words removed from the subject to work out 10 expressions of every point. Topic modeling gives a bunch of 10 words to encapsulate a subject and the theme is created by subjectively examining the collection of words introduced in the topic displaying results. Then, the most noteworthy top articles of each topic are analyzed and referred to make the theme title.

The topic coherence score compels the number of topics K and when the coherence score is the highest, K takes the optimal value. This article employs the assessment index coherence score in the statistical language model show to decide the ideal number of topics (29).

The formula for the coherence score is described as follows:


$$\begin{array}{l} coherence\;\left(V\right)=\underset{\;\left(\nu_{i},\nu_{j}\right)\in V}{\sum }score\;\left(\nu_{i},\nu_{j},\varepsilon \right)\;\\ score\;\left(\nu_{i},\nu_{j},\in \right)=\log\frac{p\;\left(\nu_{i},\nu_{j}\right)+\varepsilon }{p\;\left(\nu_{j}\right)} \end{array}$$

In the formula, V is a series of words that describe the topic. ε is the smoothing factor, which is taken as 1 according to experience. ν_i_ and ν_j_ are any two words belonging to V, respectively. p (ν_i_, ν_j_) represents the co-occurrence of ν_i_ and ν_j_ probability. The consistency score is positively correlated with the sentence similarity, as obtained by calculating the co-event recurrence of words in the sentence. Therefore, a higher consistency score could give a better result (30).

### Topic Intensity

The center element of the exploration front is “topic intensity”(31). Topic intensity addresses how much specialists focus on the study output. Higher topic attention represents higher scientific study output. From a bibliometric perspective, the number of documents in a particular field of study frontier topics is vast compared with other study topics. When identifying frontiers based on the LDA model in this article, topic intensity is recommended to reflect the great attention of the study frontier.

Topic intensity is the extent of the point in the archive. It is a quantitative pointer to quantify whether the study theme is a state-of-the-art subject. Topic intensity can be communicated as a proportion of the number of weights for exploring topics in all the scientific reports to the aggregate sum of archives. The formula is:


$$\begin{array}{l} \theta_{j}=\underset{d}{\sum }\theta_{j}^{\;\left(d\right)}/M\; \end{array}$$

In the formula, M represents the complete number of reports. θ_j_ indicates the point force value of the j subject. $\theta_{\text{j}}^{\left(\text{d}\right)}$ refers to the weight of the j topic on document d. Topic intensity can reflect the significance of the topic within the document set. The larger the topic intensity value (θ_j_) of the topic j, the more significant the proportion of the topic j in the document set and the more critical the topic j. The study frontier has the characteristic of great attention, hence the frontier topic has a relatively high proportion in the document set, with a large topic intensity value. The average topic intensity value of each topic in the document set can be calculated according to the formula. Topics with a greater topic intensity value than the average can be filtered out to obtain popular topics.

When individual topics extracted from topic modeling, the corresponding study trends of these topics over time are investigated. The weight upsides of the multitude of investigated articles were acquired and the year-to-year mean weight was charted. The exploration of preparation can be partitioned into five steps:

(1) Obtain the article data of CDW from the past 10 years.
(2) Preprocess the collected article data set to form a document set for running the LDA algorithm.
(3) Select the appropriate topic to run the LDA and get the topic and subject terms.
(4) Establish the topic intensity model, calculate the intensity of each hot topic, and analyze hot topics.
(5) According to the weight of each topic, analyze the study trend of each hot topic.

## Analysis Results

### Suitable Number of Topics

**Figure 2:**
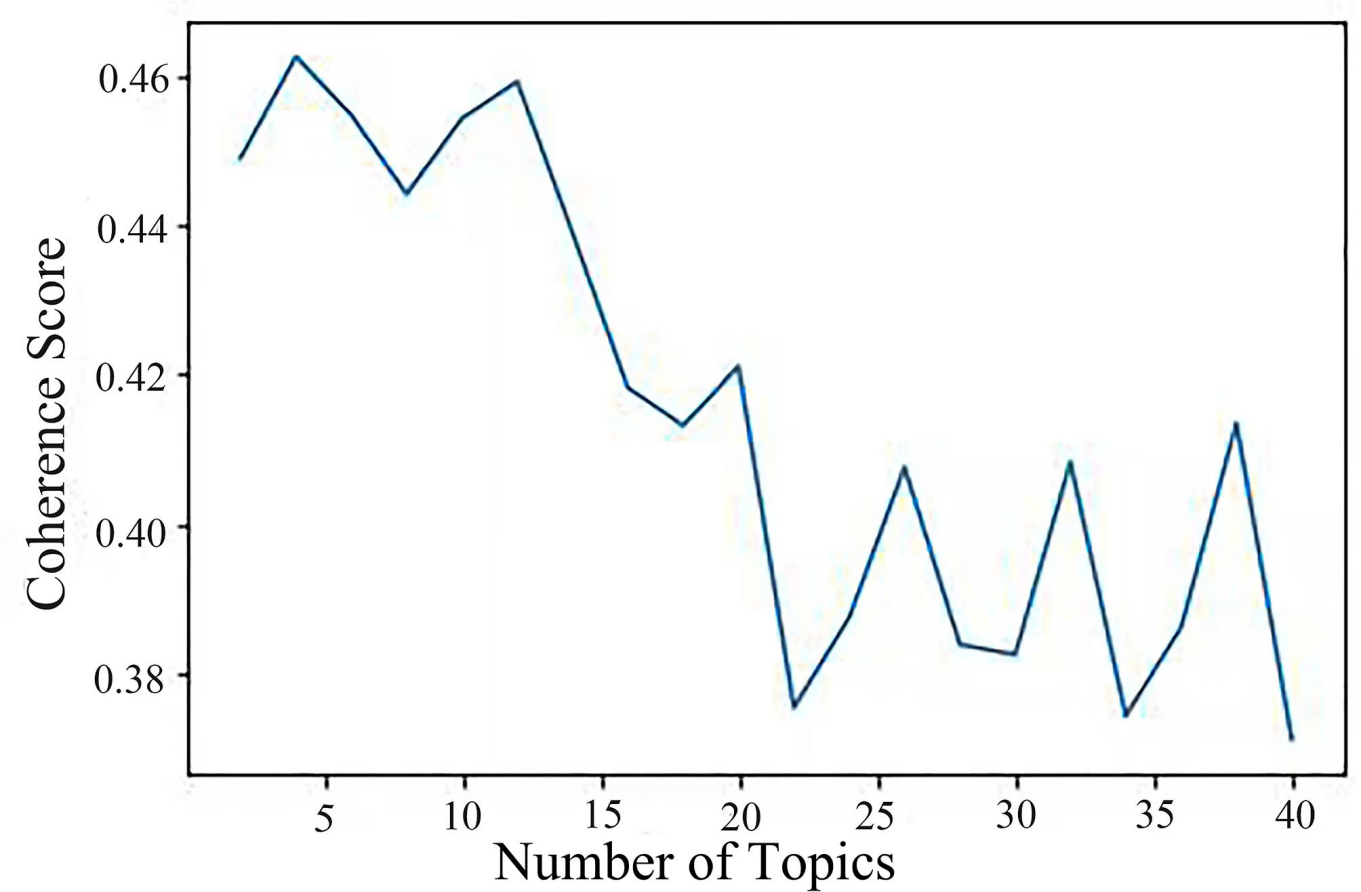
Coherence score.

Figure 2 illustrates how the number of topics varies from 2 to 40 and how coherence scores are calculated by the number of subjects. In general, a greater coherence value indicates that there are more themes that can adequately represent all of the data. As shown in Figure 2, the coherence score is the highest based on four topics. Therefore, four themes could be sufficient for this investigation.

### Making the Title of Each Topic

Python language was used to implement the LDA modeling, extract the potential topic distribution, and present it in the topic-word and document-topic distributions. After estimating the consistency score, the ideal number of points is 4. Table 1 displays the subject vocabulary distribution as a function of topic extraction. Because of spatial consideration, only the top 10 terms with the highest likelihood in each category are given in Table 1.

**Table 1:** Topic-word probability distribution.

**Topic 0**	**Topic 1**	**Topic 2**	**Topic 3**
Aggregate (0.036)	Waste (0.059)	Construction (0.021)	Material (0.016)
Concrete (0.035)	Construction (0.041)	Model (0.018)	Soil (0.016)
Recycle (0.022)	Material (0.020)	Factor (0.015)	Test (0.010)
Strength (0.020)	Demolition (0.016)	Design (0.014)	High (0.009)
Cement (0.016)	Study (0.012)	Management (0.014)	Sample (0.008)
Property (0.016)	Environmental (0.012)	Project (0.010)	Increase (0.008)
Use (0.014)	Building (0.010)	Study (0.008)	Use (0.008)
Test (0.011)	Use (0.008)	Research (0.008)	Result (0.007)
Result (0.010)	Impact (0.007)	Policy (0.008)	Brick (0.007)
Recycled (0.010)	Recycling (0.007)	Practice (0.007)	Thermal (0.007)
Recycled aggregate	Environmental impact	CDWM	Study of CDW on Soil

Table 1 presents the topics in the form of a topic-word probability distribution table. The specific topic name for each topic was summarized qualitatively according to the keywords. The final four topics are: “recycled aggregate” (topic 0), “environmental impact” (topic 1), “construction and demolition waste management (CDWM)” (topic 2), and “study of CDW on soil” (topic 3).

The LDA topic recognition generated document-topic probability matrix that is based on the probability of the 4 × 1,849 matrix. This study visualizes each document in the subject probability of topic information and intuitively investigates each document's relevant information. Table 2 presents the keywords comprising topics and the top articles of each topic and provides detailed information about each topic for easy understanding and analysis, where the Perc_Contribution column is the percent contribution of the topic in a given article.

**Table 2:** Document-topic probability distribution.

**Num**	**Perc_Contribution**	**Keywords**	**Title**
0	0.996200025	aggregate, concrete, recycle, strength, cement, property, use, test, result, recycled	Durability properties evaluation of self-compacting concrete prepared with waste fine and coarse recycled concrete aggregates.
1	0.996200025	waste, construction, material, demolition, study, environmental, building, use, impact, recycling	Construction and demolition waste management-a holistic evaluation of environmental performance.
2	0.929400027	construction, model, factor, design, management, project, study, research, policy, practice	A performance evaluation framework for construction and demolition waste management: stakeholder perspectives.
3	0.965200007	material, soil, test, high, sample, increase, use, result, brick, thermal	Spatial Distribution Characteristics of Microorganisms in Constructed Wetland System with New Matrix and Its Effect on Sewage Purification.

Four study topics reflect the current study status in the field of CDW over the past decade. Based on the words in the topic-word matrix and the top articles of each topic in the document-topic distribution, the study content in the field is analyzed and summarized, mainly focusing on the following areas:

Category I: Study on material recycling technology. The study related to material recycling technology focuses on recycled aggregate (topic 0). This category highlights material-related stability studies, which are essential for technical study on various recycled materials in CDW. In this topic, Sasanipour et al. (32) with the highest contribution rate studied the replacement of sand aggregate with recycled brick aggregate. The results showed that when using recycled brick aggregate instead of sand aggregate, chloride migration was reduced, but water absorption, drying shrinkage, and carbonation were increased.

Category II: Study on the impact of CDW on the ecosystem is the environmental impact (topic 1). This category investigates the environmental factors of CDW and proposes some measures to reduce CDW in the environment. The highest contribution rate in this topic is that Dahlbo et al. (33) used a combination of different methods to study the performance of CDW management system in Finland. In general, in most cases, the environmental benefits of waste recycling are greater than those of energy recycling.

Category III: Study on CDW management (topic 2), mainly on policy support, reduction, and production calculation. This category favors CDW management and policy study, which is closely related to stakeholders. With the highest contribution rate in this topic, Kim et al. (34) used questionnaire survey and factor analysis study methods to analyze effective strategies for providing CDW by establishing a CDW management evaluation framework. The study showed that the conceptual performance evaluation framework for CDW management consists of six factors, the key factor of which is the attitudes of stakeholders toward CDW management.

Category IV: Study on CDW in other areas, such as the study of CDW on soil (topic 3). CDW is used as a new substrate to study the ability of microorganisms in the soil to purify sewage, increasing the recycling of CDW to help protect the environment. Therefore, study in other fields is another effective way to reuse CDW resources. With the highest contribution rate in this topic, Chen et al. (35) used CDW as one of the substrates to study the spatial distribution of microorganisms and soil enzymes and the distribution characteristics of pollutants at different depths.

After obtaining the topic through experiments, the focus is on evaluating the intensity of the topic and further analyzing its hot topic. Based on the LDA topic modeling, this study uses the topic-document probability matrix obtained by the model to calculate the topic intensity, reflecting the degree of attention paid to the topic and discovering hot topics.

**Figure 3:**
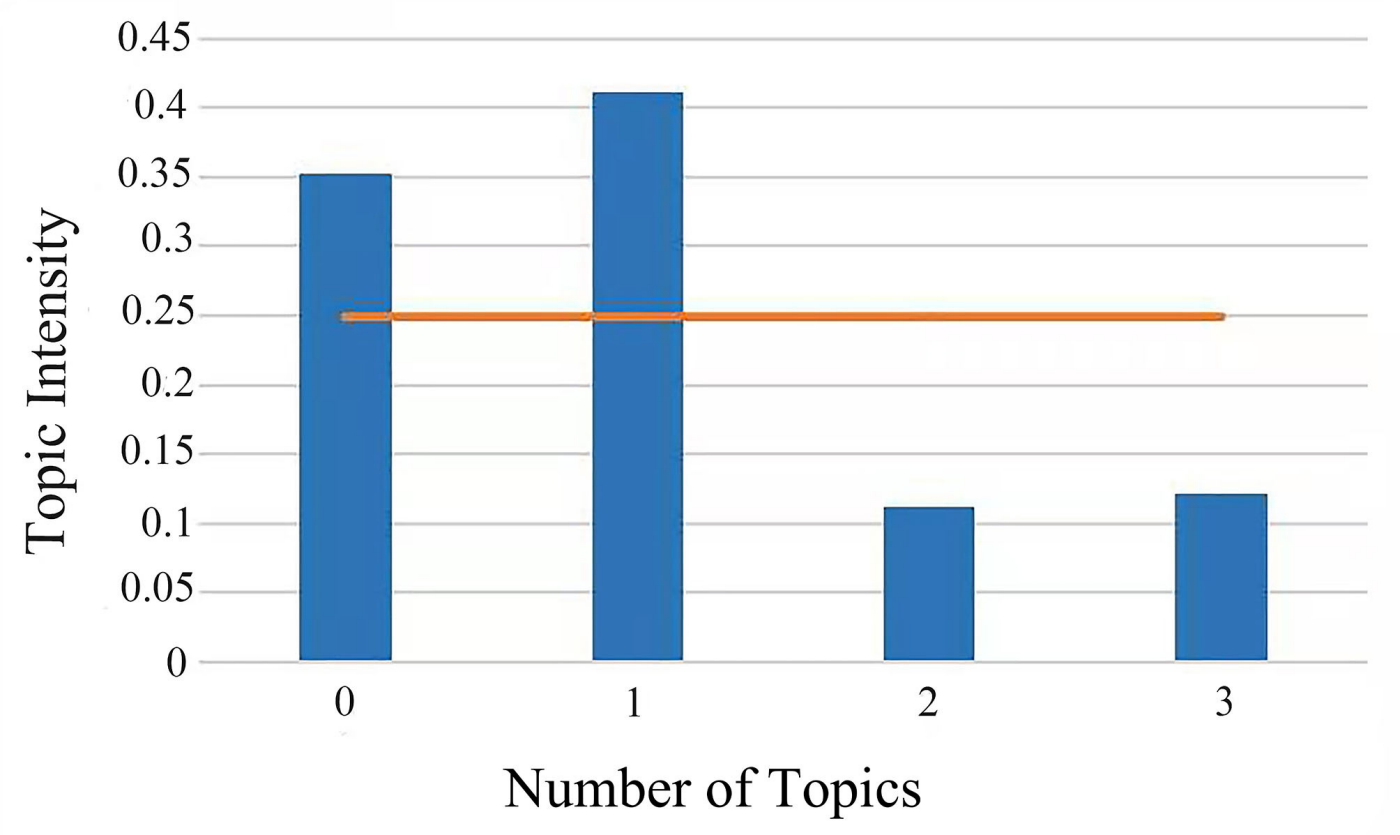
Topic intensity map.

As shown in Figure 3, the topic intensity of four topics can be calculated using the formula. The bar height represents the topic intensity value and the horizontal line in the middle is the average topic intensity value, which is 0.2491. At the same time, the topic intensity of topic 0 (recycled aggregate) is 0.3521, the topic intensity of topic 1 (environmental impact) is 0.4112, the topic intensity of topic 2 (CDWM) is 0.1118, and the topic intensity of topic 3 (study of CDW on soil) is 0.1212. Among them, topic 1 has the highest topic intensity. Furthermore, the topic intensity of topic 0 and topic 1 is higher than the average topic intensity, indicating that topic 0 and topic 1 are hotspots in CDW study field.

### Changing Interest in Each Topic Year by Year

**Figure 4:**
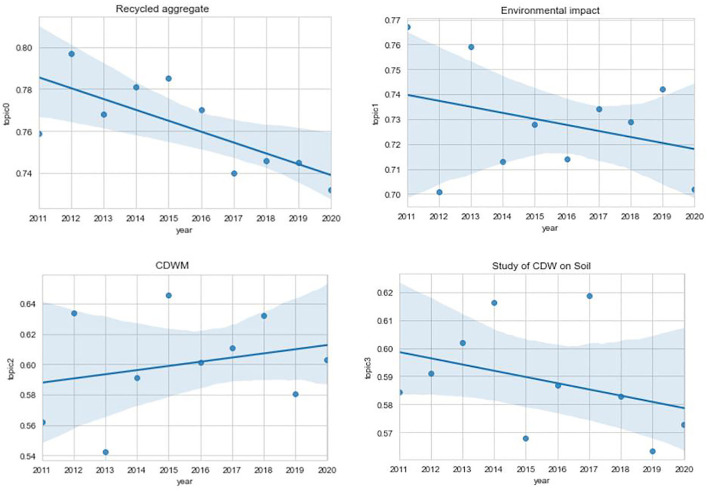
Graph of the mean weight of articles by topic.

The average annual weights of articles per topic from 2011 to 2020 are shown in Figure 4. A trend could not be discerned in this article because there were not enough data points. Consequently, the graph was redrawn with a regression line to show the trend of change.

As shown in Figure 4, only one topic (topic 2) is on the rise, representing the study on CDWM is on an upward trend. It can be seen that there is an increasing study in the field of CDW management, which may be related to the promulgation of CDW management policies in various regions in recent years. This is consistent with the findings of Li (36). In recent years, an increasing amount of study activity has been devoted to CDWM to find solutions to reduce CDW, a challenging problem around the world.

Three topics (topic 0, topic 1, and topic 3) show a downward trend. Among them, topic 0 is declining, but accounting for a large proportion of all the topics, with a weight of 0.73–0.80. This indicates that the recycling of CDW has always been an important core technology or application field. The decline in study on recycled aggregates may be due to limited technical conditions, lacking study equipment, and high costs. Moreover, there are currently fewer CDW recycling companies and limited study subjects. From the topic intensity of CDW recycling, it can be seen that its value is relatively high, indicating that the weight of CDW recycling declines, but continues to develop, which is a study hotspot. Similarly, Liu (37) found that the promotion of recycled aggregate applications mainly depends on low-cost technology and low-cost technology will be a hotspot in the future. Topic 1 is in a downward trend, but accounts for a relatively large proportion of all the topics, with a weight of 0.71–0.75. Its topic intensity is also above the average, indicating that environmental impact study is also one of the necessary study fields in CDW. Its weight declined may be because the release of CDW policies in various regions has certain particularities, study has certain limitations, and the lack of data on CDW disposal causes certain obstacles to study. At the same time, Chen (38) found that the study on the impact of CDW on the environment is still in the preliminary stage; however, the impact of CDW on the environment has gradually received more attention from researchers, which has promoted the environmental impact study of CDW. Topic 3 is also in a downward trend. It can be seen from the points that the study first rises and then falls, but its topic intensity is above the average, which indicates that the topic is still the study hotspot that year. The decline in the trend of this topic may be due to that scholars who are currently concerned about CDW are more scholars related to construction engineering. In addition, scholars in other fields pay less attention to the study on the field of CDW, which may easily lead to a decline in the study trend. Therefore, there is currently a lack of scholars who have reviewed this type of study.

### Future Study

Future study is suggested to pay attention to the impact of CDW on the environment from the perspective of the whole life cycle. The governance of CDW should be seen as a sustainable system (39). Most of the existing study is limited to the study of CDW in the construction and demolition stages (40–42), while ignoring the generation of waste in all the stages of the project life cycle. Considering the generation of waste at the design stage of a construction project, it can effectively reduce the generation of waste. In addition, the transport process may cause a high environmental load (43). Future study should pay more attention to the impact of the entire CDW life cycle on the environment to improve environmental performance.

Another future study is suggested to conduct more extensive study on different kinds of CDW. There are many types of CDW, each with different recycling routes and process modes. A single CDW processing model may be too general and not suitable for all the types of CDW. Therefore, more in-depth study on different types of CDW should be carried out, such as study on recycled glass and recycled concrete, which can provide more valuable suggestions for CDW recycling.

Apply new technologies to study related to the entire field of CDW. By analyzing the literature and combining with the current development trend, it is found that there is still a relatively lack of study on new technologies. With the rapid development of information technology, more and more new technologies have been applied to engineering construction study, such as big data, artificial intelligence, building information model, geographic information system, and so on (44–47). For example, Lee used artificial neural networks and genetic algorithms to predict the amount and cost of waste in the early stages of construction. Wu developed an innovative method using embedded geographic information system (GIS) to quantify CDW from generation to final disposal. Therefore, future study can apply intelligent technology to the entire study field of CDW and can also use two or more technologies combined for study, which will help to overcome the limitations of a single technology in application and improve the performance level.

Furthermore, CDW study can benefit from additional views, even after the findings of this study are considered. Study articles can be also expanded to incorporate additional databases [Scopus, China National Knowledge Infrastructure (CNKI), etc.]. A second option is to add earlier years to the list of publications. The history of CDW study trends is uncovered, which can shed light on the fads and features of CDW study evolving through time. In CDW field, these viewpoints provide patterns that can be used to anticipate future developments. Topic modeling is also feasible through the use of multiple algorithms, which allow for more refined or comparative identification of topics, with the potential to provide a test of the topics found. A corpus with additional metadata, such as author-offered keywords, abstracts, and titles, can be used for topic modeling. Finally, the extracted topics can also be utilized to understand and evaluate the progress of CDW study over time.

## Conclusion

Tracking the study hotspots of CDW can help scholars to understand the current study status and the law of development over time. This study proposed a method to obtain short-text topics through the combination of the LDA and topic intensity. The topics and abstracts of articles in the WoS published from 2011 to 2020 were chosen as the study subject. First, we use the NLTK and spaCy to obtain the professional terminology of CDW articles. Second, the LDA model of Mallet, a generative probabilistic topic model, was used to capture potential topics in articles of CDW to obtain the topics and topic words. Third, the topic intensity was calculated to obtain hot topics. Finally, the periodic changes of topics to year-to-year mean weight were analyzed in different periods, discussing each topic's development trend and mapping the trend of CDW study over time.

The results show that the topic effect is the best when the number of topics is four. One topic is trending up and the other three topics are trending down. Results based on the LDA have revealed these topics. Only one topic is on the rise (e.g., “CDWM”). The rise is relatively straightforward, indicating that CDWM is receiving an increasing attention in society, while others are decreasing gradually (e.g., “recycled aggregate,” “environmental impact,” and “study of CDW on soil”). Such decline may be affected by the economy, technology, and policy to a certain extent, but according to the topic intensity, it can be seen that topic 0 and topic 1 are both the hot topics. The results show the trends of CDW study and provide more valuable information for real-time, detailed, and scientific tracking of its study. The LDA can more accurately extract the study topics of CDW articles, which is helpful for researchers to have a preliminary understanding of the development status of the field, while grasping the future study direction and looking for emerging topics. Compared with the existing similar studies, previous studies have found that the study in the field of CDWM has attracted more and more scholars' attention. However, in addition to finding that CDWM has attracted much attention, this study also found that recycled aggregate and environmental impact are also hotspots in CDW study. In addition, some scholars have studied the impact of CDW on soil. It is important to consider the study's limitations when interpreting findings. First, the titles and abstracts provided by authors are studied. Previous study has focused on the article's title, abstract, keywords, and text. Titles and abstracts may not sufficiently convey the study, despite that authors are proficient in their field and in a position to do so. Second, the sample size of 1,849 articles is large, which has specific practical significance. However, only CDW articles of the WoS are considered. CDW articles published by scholars in comprehensive excellent domestic journals are not considered. Future study can consider increasing the example size for subject analysis to completely comprehend the current development status of CDW field. Third, the analysis is based on the LDA algorithm, which does not look at different calculations for point demonstration (LSI/LSA). The themes and findings of this study may vary slightly, if other topic modeling techniques are used. Fourth, the LDA model assumes that topics are independent of each other. There are always inseparable links between topics in the same discipline. Therefore, the related topic model can be added to the model in future study. Finally, this study only considers the external characteristics of the publication time. In future study, the structural topic model can be used to add external information, including authors and journal categories to understand the study status more accurately.

## Data Availability Statement

The original contributions presented in the study are included in the article/supplementary material, further inquiries can be directed to the corresponding author.

## Author Contributions

PX and ZW contributed to conception and design of the study. QH organized the database. BZ performed the statistical analysis. PX wrote the first draft of the manuscript. ZW and JZ wrote sections of the manuscript. All authors contributed to manuscript revision, read, and approved the submitted version of the manuscript.

## Funding

This study was supported by the National Natural Science Foundation of China (No. 72001148), the Foundation for Basic and Applied Basic Research of Guangdong Province (No. 2019A1515110247), the Research Start-up Funding in Shenzhen (No. 000376), and the Natural Science Foundation of Shenzhen University (SZU).

## Conflict of Interest

The authors declare that the research was conducted in the absence of any commercial or financial relationships that could be construed as a potential conflict of interest.

## Publisher's Note

All claims expressed in this article are solely those of the authors and do not necessarily represent those of their affiliated organizations, or those of the publisher, the editors and the reviewers. Any product that may be evaluated in this article, or claim that may be made by its manufacturer, is not guaranteed or endorsed by the publisher.
